# Molecular Tumor Subtypes of HPV-Positive Head and Neck Cancers: Biological Characteristics and Implications for Clinical Outcomes

**DOI:** 10.3390/cancers13112721

**Published:** 2021-05-31

**Authors:** Tingting Qin, Shiting Li, Leanne E. Henry, Siyu Liu, Maureen A. Sartor

**Affiliations:** 1 Department of Computational Medicine and Bioinformatics, Medical School, University of Michigan, Ann Arbor, MI 48109, USA; qinting@umich.edu (T.Q.); shitingl@umich.edu (S.L.); lechenry@umich.edu (L.E.H.); liusiyu@umich.edu (S.L.); 2 Rogel Comprehensive Cancer Center, University of Michigan, Ann Arbor, MI 48109, USA; 3 Department of Computer Science, LS&A, University of Michigan, Ann Arbor, MI 48109, USA

**Keywords:** human papillomavirus, head and neck cancer, cancer subtypes, gene expression, oropharynx, HPV integration, immune response, keratinization

## Abstract

**Simple Summary:**

Human papillomavirus (HPV) infections are responsible for a continually growing number of head and neck cancer (HNC) cases, with the incident rate overtaking that of HPV-related cervical cancers in the United States. Most HPV-related HNC cases arise in the oropharynx, and although they have a better 5-year survival rate than non-HPV-related HNC patients (80% compared to 50%), de-escalating treatment in all HPV(+) patients in an attempt to improve quality of life led to unacceptable results. Studying molecular subtypes of HPV(+) HNC can help to identify treatment regimens tailored to each patient’s tumor characteristics. We synthesized information from several studies of HPV(+) HNC subtypes, and describe three main groups that differ by their immune cell content, level of keratinocyte differentiation, degree of epithelial-to-mesenchymal transition, probability of HPV integration, oxidoreductase activity and stromal cell (e.g., cancer-associated fibroblast) content. The differences have important implications for local or distant recurrence, treatment response and survival.

**Abstract:**

Until recently, research on the molecular signatures of Human papillomavirus (HPV)-associated head and neck cancers mainly focused on their differences with respect to HPV-negative head and neck squamous cell carcinomas (HNSCCs). However, given the continuing high incidence level of HPV-related HNSCC, the time is ripe to characterize the heterogeneity that exists within these cancers. Here, we review research thus far on HPV-positive HNSCC molecular subtypes, and their relationship with clinical characteristics and HPV integration into the host genome. Different omics data including host transcriptomics and epigenomics, as well as HPV characteristics, can provide complementary viewpoints. Keratinization, mesenchymal differentiation, immune signatures, stromal cells and oxidoreductive processes all play important roles.

## 1. Phenotypic, Clinical and Molecular Characteristics of HPV-Positive HNSCC, and Evidence That They Are Not a Homogenous Group

With an estimated 650,000 new cases and 330,000 deaths worldwide each year, head and neck cancer represents a surprisingly heterogeneous group of tumors, the great majority of which are head and neck squamous cell carcinomas (HNSCCs) [1]. High risk human papillomavirus (HPV) infections are responsible for a continually growing number of HNSCC cases, with the incident rate now overtaking that of HPV-related cervical cancers in the United States (https://www.cdc.gov/cancer/hpv/statistics/cases.htm, accessed on 25 January 2021). Most HPV-related head and neck cases arise in the oropharynx, however a smaller percent are in the oral cavity and larynx, with negligible numbers in the other sites [2]. Worldwide, there are approximately 93,000 new oropharynx cancers cases diagnosed each year, resulting in an expected 51,000 deaths [1]. In the US, HPV is associated with approximately 36,000 new cancer cases annually, with an estimated 14,000 of those in the oropharynx (https://www.cdc.gov/cancer/hpv/statistics/cases.htm, accessed on 25 January 2021).

There is consensus among researchers that HPV-associated head and neck tumors represent a distinct tumor entity, with patient demographic, clinical and molecular differences [3,4]. Demographically, HPV-positive HNSCC patients tend to be slightly younger (mode of 60–64 years) [5], less likely to smoke, and follow a healthier diet than HPV(−) HNSCC patients [6,7]. Clinically, HPV-positive HNSCC tumors tend to be poorly differentiated and more likely to have positive lymph node status [8]. In spite of these poor prognosis markers, they have a significant survival advantage over their HPV(−) HNSCC counterparts, with an approximate 80% 5-year survival rate compared to 50% for HPV(−) HNSCC. This difference can be attributed to higher response rates to induction chemotherapy and chemoradiation [9,10]. However, this survival advantage is observed mainly in HPV(+) oropharyngeal cancer cases, with much smaller HPV survival advantages in other sites [9,11,12]. Given the high rate of survival and severe adverse effects from standard treatment protocols that significantly reduce quality of life for survivors, interest has grown in treatment de-escalation for HPV(+) oropharyngeal cancer patients. Unfortunately, two clinical trials that de-escalated treatment for all HPV(+) oropharynx cases or all with minimal smoking history resulted in worse survival [13], with both trials testing the substitution of cisplatin with cetuximab. These results suggest the need to identify the subset of patients who are most likely to benefit from de-escalation.

At the molecular level, HPV elicits initial carcinogenic hits with its two main oncoproteins E6 and E7, which target *p53* and *Rb* for degradation, respectively [14]. Disabling these key tumor suppressor proteins alone, however, is not sufficient for malignancy; several other factors, including smoking or other tobacco use history, alcohol use, patient age, patient intrinsic immune function, genetics and epigenetics also contribute to carcinogenesis. Genetically, the most commonly mutated genes in HNSCC differ depending upon HPV status. For HPV(+) tumors, top driving gene mutations include the genes *PIK3CA*, *KMT2D*, *PTEN*, *TRAF3* and *FGFR3*, and the *APOBEC* mutation signature is strongly associated with patients’ total mutational burdens [15,16]. In terms of DNA methylation, the most well-studied epigenetic mark, HPV(+) tumors tend to exhibit a hypermethylation phenotype compared to HPV(−) HNSCCs [17,18]. These factors also ultimately contribute to the heterogeneity observed within the group of HPV(+) HNSCC tumors and patient outcomes. This heterogeneity is not surprising, considering the many other important contributors to heterogeneity, including stage at diagnosis, tumor mutational profile, immune characteristics, effects of HPV integration and more.

However, despite the variations in molecular profiles and clinical outcomes of HPV(+) HNSCC patients, until recently the main research focus has been on the delineations of head and neck cancer by HPV(−)status. While many other works demonstrate the unique characteristics of HPV(+) tumors compared to HPV(−), e.g., [19], relatively few studies have examined the heterogeneity within HPV(+) tumors. As we and others have shown, such heterogeneity exists in terms of gene expression, genetics (driving mutations and copy number alterations), epigenetics (DNA methylation and 5-hydroxymethylation) and clinical outcomes, in some cases to the extent of differences by HPV(−)status. In other words, depending on the attribute studied, HPV(+) subtypes may be as different from each other as they are compared to HPV(−) tumors. Certain characteristics associated with HPV(+) tumors in general, may actually be attributable to only a subset of HPV(+) patients, and these characteristics may have important clinical effects.

Here, we review the literature on HPV(+) head and neck cancer subtypes, hoping that it will highlight the need to move the field forward from treating HPV(+) HNSCC as a homogeneous group to understanding HPV(+) HNSCC as a complex, multi-faceted group of heterogeneous cancers. We first review the early, initial recognitions of HPV(+) subtypes which were based on microarray gene expression data and thus limited in their discoveries. We then proceed to describe the defining characteristics of the main HPV(+) subtypes as fleshed out with next generation sequencing data and epigenomic assays. The HPV(+) HNSCC subtypes are then compared to HPV-associated cervical cancer subtypes, before reviewing the important role that HPV integration into the host genome plays in defining subtypes. Other mechanisms contributing to the subtypes are also discussed, before presenting what has been observed in terms of subtypes and HPV integration in relation to survival outcomes. Finally, the future potential for de-escalation treatment in a subset of HPV(+) oropharynx cancer patients is discussed, along with the potential of additional targeted therapies for subgroups with worse prognosis.

## 2. Early Research on HPV(+) Subtypes and Heterogeneity within HPV(+) HNSCCs

Studies as early as 2007 using microarray analysis were able to identify distinct subtypes in HPV(+) head and neck cancers using this gene expression data alone. In one study that included 42 head and neck cancer cases, 16 of which were HPV(+), two subgroups of HPV(+) cancers were identified after performing clustering techniques on differentially expressed genes between HPV(+) and HPV(−) cancers [20]. These two HPV(+) subgroups were consistently formulated regardless of clustering method and numbers of differentially expressed genes. Furthermore, the identified subgroups, referred to as α and β, did not significantly correlate with any particular clinical or demographic variables, such as anatomic site, age and clinical stage [20]. However, they were associated with unique pathways based on gene expression signatures. The α subgroup exhibited high up-regulation of B lymphocyte/lymphoma-related genes as well as genes expressed by endothelial cells. Genes related to small proline-rich proteins, structural cross-linking proteins of the cell envelope of keratinocytes, were down-regulated. Additionally, the α subgroup had an increased relative expression of *SYNPO2*, a gene important in regulating cell migration, which suggests that this subpopulation may have higher invasive potential [20]. The β subgroup expression pattern, conversely, suggested high keratinization (e.g., *KRT6B* and *KRT16*) and gap junction proteins for this subtype. Whether the differences in gene expression between these two subgroups reflect differences in biology and clinical outcomes remained to be determined.

A later study by Keck et al., using a microarray approach also identified two biologically distinct HPV(+) subtypes: inflamed/mesenchymal (IMS) and classical (CL) [21]. In contrast, HPV(−) head and neck cancers were molecularly categorized into three subgroups: IMS, CL and basal (BA). Despite some overlap in gene expression between HPV(+) and HPV(−) head and neck cancers within the same subgroup, many of the biological pathways associated with HPV(+) and (−) tumors remain distinct [21]. Tumors in the IMS group were characterized by expression of immune response, mesenchymal and proliferation gene signatures with downregulation of epithelial markers, and may therefore reflect tumors with CD8+ T lymphocyte infiltration, epithelial-to-mesenchymal transition (EMT) and high proliferation [21], similar to the α subgroup in Pyeon et al. This HPV subtype also had increased cell cycle pathway activities and histologically appeared poorly differentiated and non-keratinizing. Likewise, CL subtype HPV tumors also had upregulation of cell-cycle genes and proliferation signatures, but were characterized by activation of the polyamine degradation pathway [21]. However, CL subtype HPV tumors showed keratinization and were not as poorly differentiated morphologically as IMS subtype HPV tumors. Due to the use of microarray data, the relationship between HPV subtypes and characteristics of HPV itself could not be determined. However interestingly, the two molecular HPV subtypes were associated with prognosis. The IMS subtype demonstrated improved overall survival compared to the CL subtype, which may reflect the increased CD8 T cell infiltration in IMS tumors. These findings suggest that even within HPV(+) tumors, biological differences exist between tumor subtypes that may have implications on treatment and prognosis.

Studies of larger cohorts with higher resolution RNA-seq based data, genome-wide DNA methylation and HPV insertion sites into the host genome helped to further subclassify HPV(+) tumors and identify causal factors for the differing expression patterns. The first of these was from The Cancer Genome Atlas (TCGA) HNSC project. Using RNA-seq data for 36 HPV(+) and 243 HPV(−) head and neck cancer tumors, the TCGA HNSC project identified four gene expression subtypes: atypical, basal, classical and mesenchymal [16]. However, almost all HPV(+) samples were classified as atypical, which could be due to HPV(−) samples driving the subtype analysis, as there were many more HPV(−) samples than HPV(+). Overall, The TCGA analysis could not properly distinguish HPV(+) subtypes, but following their project, multiple RNA-seq based studies were able to identify subtypes within HPV(+) HNSCCs, often utilizing the HPV(+) TCGA samples which increased in number over time. Table 1 provides an overview of the main microarray and RNAseq studies used to define HPV(+) HNSCC subtypes.

## 3. Defining Characteristics of the Main HPV(+) Subtypes

**Figure 1:**
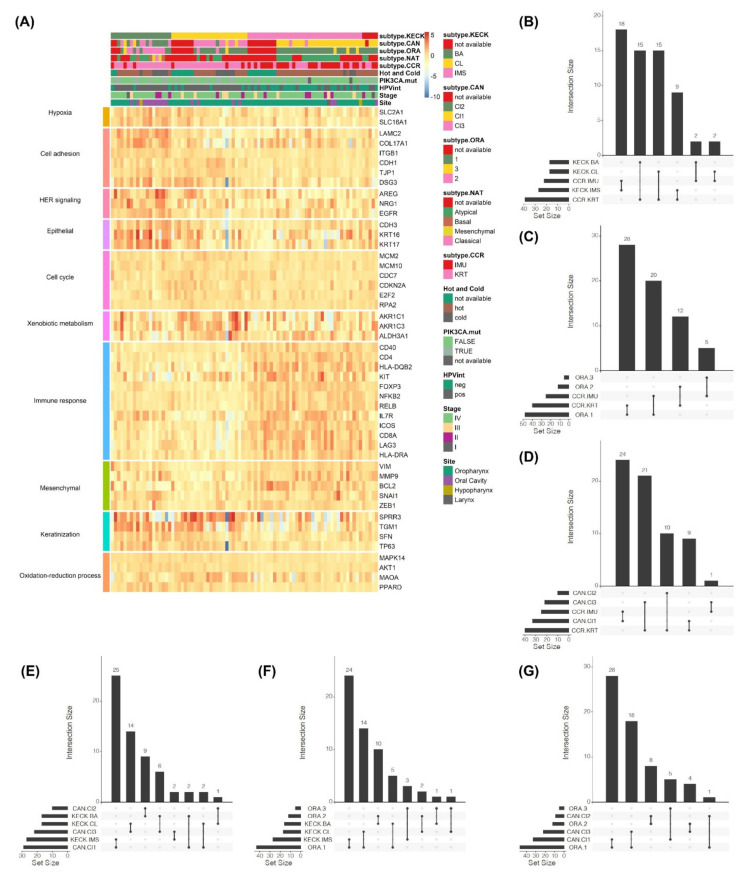
Comparison of the different definitions of HPV-positive head and neck subtypes: (**A**) Heatmap illustrating expression of genes and pathways previously identified as distinguishing HPV(+) subtypes; genes and pathways were combined from Keck et al. (KECK) and Zhang et al. (CCR) subtype findings, and visualized using log2FPKM values normalized (mean centered) by genes and samples. Additionally, shown are several annotations which indicate trends among subtypes and with tumor characteristics. KECK subtypes were re-designated for all 66 TCGA and 18 UM HPV(+) cases by applying their algorithm, while all other subtype definitions were obtained directly from the original publications. (**B**–**G**) Upset plots illustrating pairwise overlaps among subtype definitions: (**B**) KECK (BA/CL/IMS) vs. CCR (IMU/KRT), (**C**) ORA (1/2/3) vs. CCR (IMU/KRT), (**D**) CAN (Cl1/Cl2/Cl3) vs. CCR (IMU/KRT), (**E**) CAN (Cl1/Cl2/Cl3) vs. KECK (BA/CL/IMS), (**F**) ORA (1/2/3) vs. KECK (BA/CL/IMS) and (**G**) ORA (1/2/3) vs. CAN (Cl1/Cl2/Cl3).

During the last five years, multiple studies have uncovered new evidence further characterizing the main HPV(+) HNSCC subtypes, the main ones being Zhang et al., Locati et al. and Lee et al. [22,23,24]. Based on gene expression profiles of a combination of 18 HPV(+) HNSCC from University of Michigan Hospital and 66 TCGA HPV(+) HNSCC samples, Zhang et al., revealed two HPV(+) subtypes, which were named IMU and KRT. Similar to the α and IMS subgroups described above, the IMU subtype was defined by a heightened immune response, mesenchymal differentiation and angiogenesis expression, whereas the KRT subtype was identified by stronger keratinization [22] (expression level shown as subtype.CCR in Figure 1A). By using RNAseq data, they were able to identify HPV integration breakpoints in the genome and predict HPV integration status. Studying this in relation to subtype led to the novel finding that the KRT subgroup was much more likely to harbor viral integration events than IMU (indicated by HPVint in Figure 1A). The study also linked the IMU subtype with stronger epithelial-to-mesenchymal transition (EMT) signatures and higher expression of *BCL2*, an anti-apoptotic regulator associated with resistance to chemotherapy and radiation. By retrieving gene expression from 11 HNSCC studies, Locati et al. characterized three distinct HPV(+) subtypes, defined as Cl1 (immune-related), Cl2 (highly keratinized, epithelial mesenchymal transition-related and hypoxia), and Cl3 (highly keratinized, proliferation-related) [23]. The Cl1 subtype was in agreement with the IMU subtype, while the KRT subtype was further stratified into Cl2 and Cl3 by their biological and prognostic characteristics (indicated as subtype.CAN in Figure 1A). A different approach to defining HPV(+) HNSCC subtypes was taken by comparing them to cervical, lung and esophageal tumors, which each have similarities to HNSCC. Instead of naming subtypes after their most distinguishing gene signatures, Lee et al., did so according to their similarity to other tumor types [24]. The three HPV(+) subtypes were called Subtype 1 (cervical-like; 22% HNSCC; activated Protein Kinase A signaling, Vascular endothelial growth factor (VEGF) signaling, mechanistic Target Of Rapamycin (mTOR) signaling and IL-8 signaling), Subtype 2 (HNSCC-classical; 91% HNSCC; activated RhoA, PI3K/AKT and NF-kB signaling pathway), and Subtype 3 (lung-like; 11% HNSCC; activated nicotine degradation, Notch signaling, Xenobiotic metabolism and Wnt/β-catenin signaling; basal cell active in cell cycle pathways), which are marked as subtype.ORA in Figure 1A. Even though most HPV(+) tumors (74%) fell into the cervical-like subtype, a significant fraction were designated as either lung or HNSCC-classical.

Together, these studies revealed several genetic subtype differences in HPV, copy number alterations and cancer gene mutations. In the classical subtypes, Keck et al., discovered frequent amplification of *E2F3* (6p22), which encodes a transcription factor important for cell-cycle regulation and DNA replication [21]. Consistent with the KRT subtype being more likely to have HPV genic integration than the IMU subtype, the KRT group also showed lower expression of the viral genes E2, E4 and E5, the expression of which is often lost upon integration. By analyzing tumor and blood single nucleotide polymorphism (SNP)-array data from the same cohorts, the authors were able to identify more amplifications in KRT tumors than IMU, especially at chromosomal arms, and being compared with the results from gene expression suggested that the gain and loss of copy numbers can partially drive the expression differences between the two subgroups. In terms of the differences in gene mutation frequencies between the two HPV(+) subtypes, analysis of non-synonymous mutations revealed only one gene with a difference of more than 20% between two groups, which occurred on oncogene *PIK3CA* in 37% of the KRT samples and only 16% of the IMU samples. *PIK3CA* activating mutations are able to enhance mTOR activity and inhibit autophagy in HPV(+) HNSCC, leading to the predisposition of these cancer cells to avoid autophagy, ferritinophagy and ferroptosis [25,26]. HPV(+) head and neck tumors with high mesenchymal expression, which includes the IMU/IMS subtype and a subset of the highly keratinized group (Cl2 subtype with high stromal and EMT), exhibit upregulation of lipid peroxidation [27], resulting in higher sensitivity to ferroptosis [26]. The *PIK3CA* activating mutations may therefore be a mechanism to avoid cell death by ferroptosis in these tumors, especially in the highly keratinized, high stromal and high EMT tumors which more often have *PIK3CA* mutations. The study by Lee et al. also characterized the mutation landscape of Subtype 1 (infrequent *TP53* mutation and *PIK3CA* amplification), Subtype 2 (*TP53* mutation, *CDKN2A* deletion and *NOTCH* alteration) and Subtype 3 (*TP53* mutation *CDKN2A* deletion, *PIK3CA* amplification and high mutation rate of *AJUBA*, *MUC17*, *KMT2D* and *NFE2L2*). The *PIK3CA* mutation status is also marked in Figure 1A.

Within HPV itself, the shorter isoforms of HPV 16 oncogene E6 (E6*) are known carcinogenic factors in HNSCC, and Zhang et al. identified that the HPV16-KRT subgroup is associated with higher E6* levels (defined as the percent of E6 expressed as E6*) than the IMU subtype [22]. Full length E6 and E6* were shown to bind to procaspase 8 at different domains, which has important implications for how they inhibit caspase 8 dependent apoptosis, necroptosis and caspase 8-E6 binding inhibitors to resensitize the tumor to apoptosis inducers [28]. In addition, a 2020 study developed an influence score of HPV16 E6* (the shorter isoforms of E6) and identified its significant association with carcinogenic pathways, tumor size and survival in HPV(+) HNSCC, suggesting that the E6* influence score can potentially serve as a prognostic factor for those patients [29]. This paper also found HPV integration (+) patients exhibited significantly higher E6* influence score than HPV integration(−) patients (Wilcoxon rank-sum test, *p* = 0.02), which is in line with the previous finding based on subtypes.

**Figure 2:**
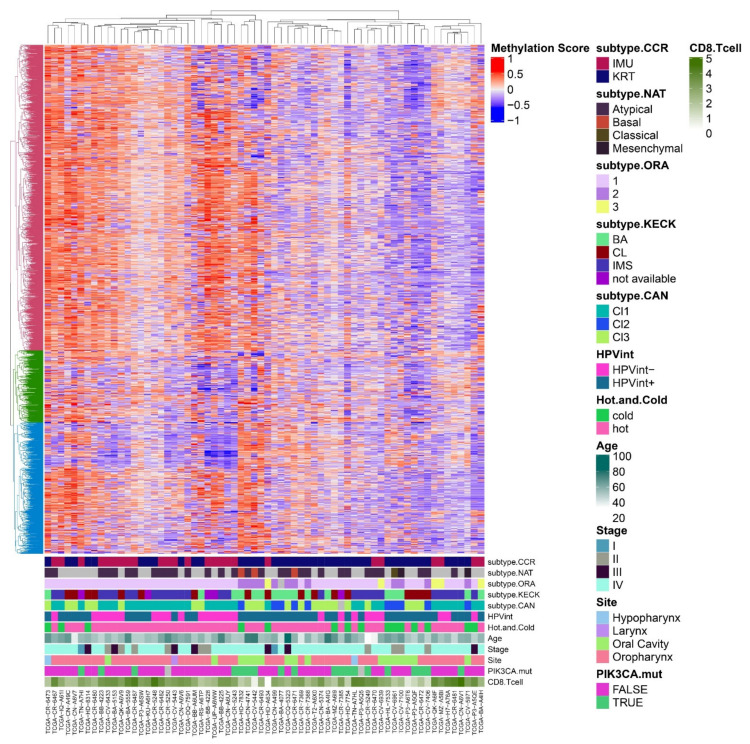
DNA methylation profiles associate with HPV(+) HNSCC subtype. Heatmap of DNA methylation data (normalized beta values) assessed by the Illumina 450K BeadChip for 66 HPV(+) HNSC TCGA samples, and annotated with subtypes and additional tumor characteristics. The top 1% most variable CpGs (probes) were used based on standard deviation. The distance matrix was calculated using Euclidean distance, and hierarchical clustering was performed using complete linkage. Cluster colors on left were determined by applying consensus clustering and selecting the optimal number of clusters.

The HPV(+) subtypes also exhibit distinct epigenetic profiles. Differences between the two subtypes IMU and KRT were strikingly observed in a 2020 epigenetic study of the University of Michigan HNSCC cohort capturing the genome-wide 5-hydroxymethylation (5hmC) profiles [30]. The 5hmC epigenetic mark is the first step in the demethylation pathway and results in a loss of transcriptional repression in promoters and enhancers, often serving as a mechanism to activate differentiation and developmental programs [31]. In the above 5hmC HNSCC study, the IMU subtype was easily distinguished from the KRT HPV(+) and HPV(−) samples, whose 5hmC profiles overlapped with each other. Overall for HPV(+) tumors, global 5hmC levels were elevated in the KRT subtype compared to IMU, which is consistent with the more differentiated nature of KRT, and the 5hmC level on promoters alone was sufficient to distinguish these two subtypes [30]. These results should be interpreted in light of the fact that 5hmC is depleted in various cancer types [32,33,34], however higher 5hmC has been associated with aggressive tumors and worse survival outcomes specifically in oral cancers [35]. The epigenomic profiles in IMU and KRT highlighted extensive differences in regulatory marks at keratinocyte enhancer regions, and in the regulation of cell junction, migration and immune genes [30]. Based on previous evidence that DNA methylation patterns of HPV(+) HNSCC were involved in the activity of HPV oncoproteins (one of the key factors in identification of HPV(+) subtypes) [36], we hypothesize that the IMU and KRT subtypes could be differentiated by their DNA methylation profiles as well. Even though no published study has examined DNA methylation in HPV(+) subtypes directly, visualizing the TCGA methylation data from Infinium® 450K BeadChip data (Illumina Inc, San Diego, CA, USA) (using the 1% most variable probes) reveals two main patient groups of approximately equal number distinguished by overall high vs low methylation levels. In relation to the previously-defined subtypes, these DNA methylation clusters demonstrate clear trends that the HPV(−)IMU (subtype.CCR), Subtype 1 (cervical-like in subtype.ORA), HPV integration-negative (HPVint-), Cl1 (immune strong in subtype.CAN) and “hot” tumors (hot and cold) are associated with higher methylation levels overall (Figure 2).

Deconvolution of tumor cell type composition can also be performed with genome-wide DNA methylation data, revealing patterns of immune cell infiltration. This type of analysis was conducted using the MethylCIBERSORT method on TCGA HNSCC samples, which were classified as either immune “hot” (more T-lymphocyte infiltration) or “cold” [37]. Even though most HPV(+) tumors were classified as “hot”, nearly all of the “cold” HPV(+) tumors were of the KRT subtype and were HPV integration(+) (marked as hot and cold in Figure 1A and Figure 2). These two or three HPV(+) subtypes described above may also carry implications for survival and prognosis, which is discussed in further detail below.

## 4. Comparisons of the Identified Subtypes in HPV(+) HNSCC

Multiple studies have investigated HNSCC transcriptional profiles using the TCGA-HNSC cohort and identified varied subtypes by un-supervised clustering, including Atypical/Basal/Classical/Mesenchymal subgroups, referred to as “NAT” hereafter [16,38], BA/CL/IMS clusters, referred to as “KECK” [21], IMU/KRT HPV(+) subtypes, referred to as “CCR” [22], and clusters 1–3, referred to as “ORA” [24]. The five studies clustered different subsets of the TCGA expression data. In all studies except KECK subtypes, we used the original results published. For KECK subtypes, we reimplemented their algorithm on 84 HPV(+) HNSCC patients, including the TCGA HPV(+) HNSC cohort. Several tumors were classified as BA which differs from their original publication which had only HPV(−) samples belonging to the BA subtype (Figure 1A). By overlapping the identified subgroups of those HPV(+) samples, we found that ~83% (30/36) of HPV(+) samples were NAT Atypical [16], among which one half were CCR IMU (immune strong) and the other were CCR KRT (highly keratinized) subtypes. This suggests the TCGA clustering of the 279 TCGA-HNSC samples were driven by HPV(−) samples, and the HPV(+) samples mainly fall in the Atypical subgroup without preference to the IMU or KRT subtype.

As compared to the KECK subtypes, the majority of CCR IMU samples (18/22, ~82%) were assigned to the IMS cluster (immune strong), and the KRT samples were split between BA (basal-like) (15/39, ~38%) and CL (classical) (15/39, ~38%) clusters (Figure 1B). The ORA cluster 1 (cervical-like) subgroup contained the great majority of HPV(+) tumors and was a mixture of CCR IMU (28/48, ~58%) and KRT (20/48, ~42%) subtypes, however the 12 samples in ORA cluster 2 (HNSCC-classical) were all KRT, and the 5 in cluster 3 (lung-like) were all IMU (Figure 1C). The comparison between CCR and CAN subtypes showed that nearly all CCR IMU samples were in CAN Cl1 (immune strong; high stromal) (24/25, ~96%), and all of the 10 CAN Cl2 samples (highly keratinized; high stromal) and 21 out of the 22 (~95%) Cl3 (highly keratinized; low stromal) samples were CCR KRT subtype (Figure 1D). Moreover, the overlaps among KECK and CAN subgroups demonstrated that the KECK and CAN immune strong subtypes had high overlap (KECK IMS samples were mainly CAN Cl1 (25/27, ~93%)), and that KECK and CAN split the highly keratinized samples similarly: KECK CL were mainly CAN Cl3 (low stromal) (14/16, ~88%)) while nearly all CAN Cl2 (high stromal) were KECK BA (9/10 (90%)) (Figure 1E). The majority of KECK IMS and CL subtypes were ORA cluster 1 (cervical-like) (24/27 (~89%) and 14/26 (~88%), respectively), while over half of KECK BA was ORA cluster 2 (10/16, ~63%) (Figure 1F), and similarly, CAN Cl1 (28/31, ~90%) and Cl3 (18/22, ~82%) subtypes (both high stromal subtypes) were mostly ORA cluster 1 (cervical-like), and almost all CAN Cl2 (highly keratinized; high stromal subtype) (8/9, ~89%) was ORA cluster 2 (HNSCC classical) (Figure 1G).

These findings demonstrate that the subtypes identified by different studies although highly overlapping are not completely concordant, indicating how the input data and analysis pipeline impact the subtype definition by prioritizing different pathways. Of these five studies, only Locati et al., defined the subtypes using only HPV(+) samples, whereas the subgroups identified by TCGA and Lee et al., were mostly driven by HPV(−) samples. These differences in cohorts likely contributed to differences, partially confounding HPV(+) subtype definitions with HPV status tumor characteristics. Over 80% of basal (KECK BA: 16/19) and classical (KECK CL: 20/24) subtypes from Keck et al., were identified as HPV integration(+), and all highly keratinized/high stromal samples (CAN Cl2: 10/10) and ~77% of the high keratinized/low stromal subtype (CAN Cl3: 17/22) from Locati et al., were HPV integration(+). It is worth noting that out of the HPV(+) samples, over half of the BA cluster were oral cavity HNSCC (~56%), whereas CL (~83%) and IMS (~94%) samples were nearly all oropharynx (Fisher’s exact test (FET), *p* = 0.0002); and over half of IMS (~67%) samples were HPV integration(−), a significantly lower percent than the other two subtypes (FET, *p* = 2.86 × 10^−5^) (Figure 1A). Similarly, the ~42% of CAN Cl1 (immune strong) samples that were HPV integration(+) is significantly lower than for the other two subtypes (FET, *p* = 5.02 × 10^−4^) (Figure 1A).

By synthesizing the findings of these studies, we arrive at three overall subtypes: (1) an immune strong subtype derived from mainly oropharynx tumors with no detected HPV integration, higher mesenchymal differentiation and high stromal content; (2) a highly keratinized, yet basal-like with high stromal content subtype that are most likely HPV integration(+) and more likely to be from oral cavity primary tumors, with more classical HNSCC expression signatures (as compared with lung or cervical); and (3) a highly keratinized subtype or oropharynx tumors with low stromal content also most likely to be HPV integration(+). However, a subset of immune strong tumors may have more lung cancer-like expression signatures with worse survival, as suggested by Lee et al.

## 5. Relationship to Cervical Cancer Subtypes

Since HPV(+) head and neck cancers have driving mutations and cancer pathways in common with cervical cancers, including *PIK3CA*, *FAT1*, *CASP8*, *PTEN*, etc. [39], comparing the subtypes of HPV(+) HNSCC with those of cervical cancer may reveal additional insights. Cervical tumors have histologically been characterized by adenocarcinoma (originating from glandular cells) and cervical squamous cell carcinoma (CSCC), in which CSCC accounts for ~80 to 90% of cases. Regardless of the histological subtypes, ~95% of cervical cancers are caused by persistent infection with carcinogenic HPV (mostly HPV16) [40], and substantial heterogeneity exists within HPV(+) CSCC. Lu et al., revealed two subtypes of HPV16(+) CSCC, HPV16-IMM and HPV16-KRT, by supervised clustering of immune signatures followed by pathway analysis to identify the keratinization in the weak immune group [41]. Similar to the IMU/IMS subtype in HPV(+) HNSCC, the HPV16-IMM exhibited a strong immune response and mesenchymal features, whereas HPV16-KRT was characterized by elevated expression of genes in keratinization, biological oxidation and Wnt signaling, which is comparable with KRT-HNSCC. Similarly, Wnt/β-catenin signaling was found to be upregulated in HNSCC cluster 3 by Lee et al. [24], which has been studied as a target pathway in many cancer treatments [42]. It is noted that the HPV16-IMM-CSCC demonstrated a significantly better overall survival (log-rank *p* = 0.017, HR = 0.3) and progression-free survival (log-rank *p* = 0.035, HR = 0.7) than HPV16-KRT-CSCC. A significant difference in overall survival was also observed in the HPV(+) HNSCC subtypes identified by Locati et al.,: Cl1 (immune strong) showed the best outcome, Cl2 the worst and Cl3 an intermediate survival rate (log-rank *p* = 4.79 × 10^−9^). Concordantly, both IMU-HNSCC and HPV16-IMM-CSCC have favorable prognosis, as compared to their KRT counterparts. However, Lu et al.. failed to identify a significant association of HPV integration status with a subtype, as shown for the KRT subgroup of Zhang et al. [22]. These findings indicate that HPV infection may induce similar mechanisms of malignant transformation in both HNSCC and CSCC, resulting in two different subtypes (IMU/KRT), although some distinct factors may exist in each entity.

## 6. Dominant Role of HPV Integration in Defining HPV(+) Tumor Characteristics and Subtypes

### 6.1. Approaches to Detect HPV Integration

One potential causative mechanism identified for explaining the differentiating characteristics of HPV(+) tumor subtypes is integration of the HPV oncogenes into the host genome. Multiple direct capturing integration event techniques and indirect computational algorithms for high-throughput sequencing data have been designed to detect or quantify HPV integration events and their insertional breakpoints. Which of these methods best correlate with HPV(+) tumor subtype and/or survival, however, is unknown. Amplification of papillomavirus oncogene transcripts (APOT) is a technique focusing on the detection of viral host fusion RNA transcripts, which is sensitive to distinguishing episome versus integration-derived HPV mRNAs [43]. An alternative approach to measure the integrated HPV gene transcript activity makes use of RNA-seq data, along with several software programs for sensitively capturing the specific insertion sites, and the associated genes [44,45]. However, a high-quality RNA requirement discourages those methods from being utilized with RNA retrieved from challenging, potentially highly-degraded specimens, such as from formalin-fixed and paraffin-embedded (FFPE) blocks [46].

Complementary to the RNA approach, and avoiding the difficulties of easily degradable RNA, is to measure DNA instead. Examples of this include the Detection of Integrated Papillomavirus Sequences (DIPS) assay, DNA FISH (Fluorescence in situ hybridization), quantitative and real time PCR, MLPA and DNA-seq [46,47,48,49,50]. Each DNA assay has its own limitations, for instance, DNA FISH is designed specifically for paraffin-embedded tissue, whereas quantitative PCR measures the relative E2 gene loss compared to E6, which may miss integration events that occur outside the E2 gene region. Nowadays, more studies focus on distinct DNA-seq techniques to capture HPV integration events, having shown to be more accurate and comprehensive [51,52]. However, whereas RNAseq methods may miss integration events that are transcribed but do not result in any viral-host fusion transcripts, the DNA approaches by their very nature cannot determine which integration events are transcribed. Even though DNA validation is sufficient to determine disruption of a gene function, RNA expression is needed if the goal is to understand the general, non-site-specific effects of HPV integration on cancer pathways, such as lymphocyte activation, keratinization, mesenchymal differentiation or oxidative phosphorylation. In summary, the most thorough way to study HPV integration is to combine RNA and DNA techniques, for example, Olthof examined HPV integration on 75 OSCC patients with both DIPS and APOT [53], and Ziegert et al., applied DIPS and APOT on anogenital lesions to study the integration loci [54]. Such designs allow both comprehensive DNA integration and transcriptional detection [55].

### 6.2. The Consequences of HPV Integration

In addition to its impact on viral gene expression, HPV integration has direct and indirect effects on host gene expression. The nonrandom appearance pattern of the integrated sites across the human genome discovered by many studies strongly indicate integration events influence HNC development [56,57]. Walline et al., used DIPS to identify HPV insertion sites and found that 7 of 9 HPV(+) cell lines exhibited integrations in cancer-related genes including *TP63*, *DCC*, *JAK1*, *TERT*, *ATR*, *ETV6*, *PGRP*, *PTPRN2* and *TMEM237*, which indicates integration is a potential carcinogenic driver [56]. Another study from 84 HPV(+) HNSCC RNA-seq revealed that integration events are overrepresented in genes often mutated in head and neck, lung, and urogenital (e.g., cervical) cancers, which included *CD274*, *FLJ37453*, *KLF12*, *RAD51B* and *TTC6* [57]. The discovery that insertion events are overrepresented in these cancer-related genes also indicates that there is a natural selection of tumor cells with breakpoints in or near HPV(−)associated HNSCC-relevant genes.

In addition to directly shifting host target gene expression, HPV integration also affects the DNA methylation of the host genome. Parfenov et al., revealed highly different DNA methylation profiles for samples with versus without HPV integration events, and reported four critical differentially methylated genes, two of which hypermethylated are tumor suppressors, *BARX2* and *IRX4*, and two others hypomethylated being related to tumorigenesis, *SIM2* and *CTSE* [58].

Since integration is not a normal process in the HPV replication cycle, it often results in partial deletion of the viral genome but maintains the main oncogenes E6 and E7 [59]. High E6 and E7 expression occurs in the early phase of normal HPV replication, which yields host cell growth, differentiation inhibition and chromosomal instability. Those effects contribute to carcinogenesis, and lead to the cells continuing in the basal or partially differentiated condition, which is also a distinguishing characteristic between the IMU and KRT subtypes [60,61,62]. Thus, HPV integration and loss of episomal HPV expression has strong indirect effects on expression of keratinocyte differentiation genes and could explain much of the differentiation differences observed between the IMU and highly keratinized subtypes.

The expression of important shorter isoforms of the E6 gene, collectively called E6* are also associated with HPV integration and have been found to correlate with HPV(+) tumor subtype classification, with higher relative E6* expression in the KRT subgroup. One study showed that higher E6* protein concentration increases the level of reactive oxygen species (ROS), inducing the oxidative stress mechanism resulting in heightened DNA damage [63]. Those two pathways have been shown as key factors separating IMU and KRT subtypes. In Qin et al., this DNA damage was further shown to be associated with a higher mutational burden [29], and a higher ratio of E6* to full length E6 impact score was associated with larger tumor size at diagnosis and worse overall survival, further suggesting unfavorable prognosis for HPV integration(+) patients.

A comprehensive network describing the relationship between E6, E6* and E7 oncoproteins with HPV integration remains to be fully understood based on the current studies. An increasing number of studies have verified that E7 suppresses the antitumor immune response by silencing important genes and pathways such as *CXCL14* and STING DNA-sensing [64,65,66], which is also a main differentiating factor between IMU and KRT, and KECK subtypes. A reasonable hypothesis for the HPV oncoproteins network is that HPV integration activates E6* expression, which has been shown to stimulate the translation of E7, resulting in increased immune suppression, oxidative phosphorylation and oxidative stress. Therefore, E6* may play a key role in leading to HPV (+) tumor subtypes.

## 7. Possible Contributors to Defining HPV(+) Tumor Subtypes Besides HPV Integration, and Potential for Defining Finer-Grained Subtypes

**Figure 3:**
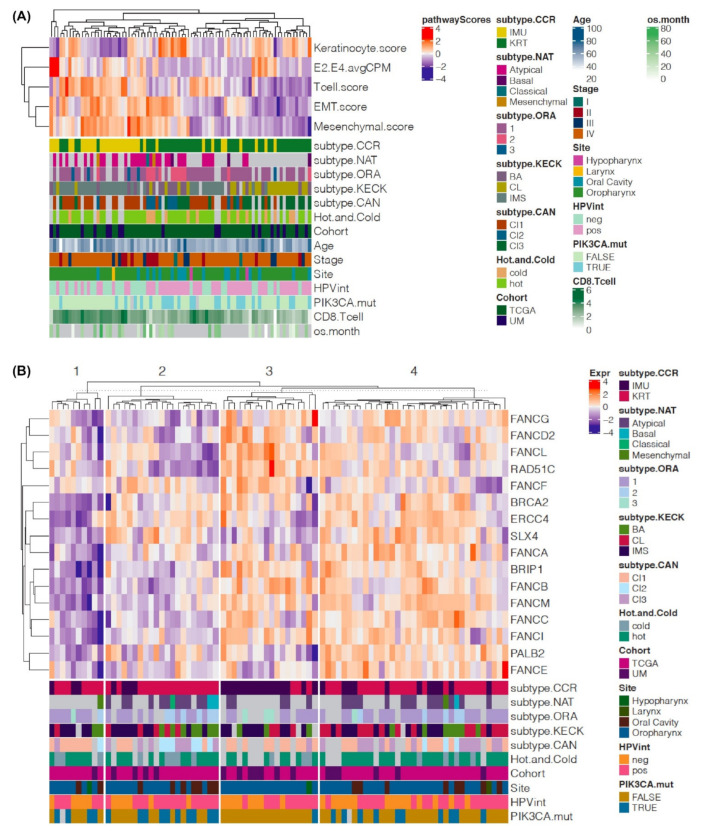
Clustering of the defining pathway scores and HPV scores visually distinguish the main HPV(+) subtypes. (**A**) Hierarchically clustered heatmap showing averaged HPV16 E2 and E4 expression (E2.E4.avgCPM) and pathway scores of keratinocyte differentiation (Keratinocyte.score:), T cell differentiation (Tcell.score), epithelial-mesenchymal transition (EMT.score) and mesenchymal cell differentiation (mesenchymal.score) among the 84 HPV(+) HNSCC samples from combined TCGA and UM cohorts. (**B**) Clustered heatmap based on genes in the Fanconi Anemia (FA) pathway. The 84 HPV(+) samples were clustered by K-means clustering (K = 4) based on the expression of 16 FA genes.

As described above, HPV integration is significantly associated with the identified subtypes in HPV(+) HNSCC, where the strongly keratinized subgroups (KRT, Cl2 and Cl3) tend to have more HPV integration events than the strong immune subgroups (IMU and Cl1) (Figure 3A) [22,24,57]. However, the subgroups still show heterogeneous HPV integration status: out of the 66 HPV(+) HNSCC in TCGA cohort, 36% (9/25) of IMU have HPV integration, and 22% (9/41) of KRT samples had no identified HPV integration events (Figure 3A). One possible explanation for this heterogeneity is that some integration events were incorrectly assigned, especially in samples with low E2 and/or E4 gene expression that were assigned as HPV integration negative. In these cases, an insertional site may have been missed; however, upon inspection we did not see any case with low E2 or E4 expression relative to E6 and E7 that was assigned to be HPV integration(−). In addition, some samples classified as HPV integration positive may actually be mixed, having both episomal HPV copies and integrated expressed E6 and E7. To seek other possible contributors to the HPV(+) HNSCC subtypes, we visualized relevant supporting data together for each patient. Specifically, we calculated the pathway scores for the 84 HPV(+) HNSCC samples (18 UM SPORE and 66 TCGA) by summarizing the expression levels for the pre-defined gene sets (pathways) as described in [22], which measure the immune response (“Tcell.score”, “CT8.Tcell”, and scores for “Dendrite cell marker”, “immune marker for hnscc” [67]) and cell differentiation (“keratinocyte.score”, “Mesenchymal.score” and “EMT.score”), and correlated the scores with IMU/KRT, IMS/BA/CL, and other subtypes (Figure 3A). In line with the previous findings [22,57], overall IMUs showed lower keratinocyte but higher mesenchymal and immune scores, whereas KRTs showed lower mesenchymal but higher keratinocyte scores, independent of the HPV integration status (Figure 3A). The mesenchymal/EMT and immune scores in HPV integration(+) IMUs tend to be lower than those in HPV integration(−) IMUs, although the difference is not statistically significant; what distinguishes them clearly from the KRTs is their overall lower keratinocyte score and higher mesenchymal/EMT. This reminds us that although HPV integration frees the tumor cells from the partial-differentiation program required to maintain the episomal HPV lifecycle, not all tumors with HPV integration become highly keratinized. To see why some HPV integration(−) samples were classified as KRT, we first noted that the immune scores and the E2/E4 expression in HPV integration(−) KRTs are actually comparable to those in IMU and significantly higher than those in HPV integration(+) KRTs (Figure 3A and Figure S1); what distinguishes these samples clearly from the IMU subtype is their overall higher keratinocyte score and lower mesenchymal/EMT. The findings suggest that the IMU/KRT subtypes are mainly directed by the cell differentiation status, and HPV integration attenuates the immune response and reduces the EMT in HPV integration(+) IMU HNSCC.

Fanconi Anemia (FA) is a known predisposition to HNSCC, and has been identified to be downregulated in sporadic HNSCC [68]. Qin et al., identified that HPV(+) patients had more Nonsynonymous, Rare and Damaging (NRD) expressed variants in FA genes than HPV(−) patients [69]. We also found FA genes were significantly downregulated in KRT as compared to IMU (Wilcoxon test, *p* = 0.002), especially in HPV integration(+) KRT (Figure 3B, Figure S2A,B), and KRTs tended to have more mutations in FA genes (FET, *p* = 0.06). Interestingly, the distribution of the 4 HPV types (HPV16, 18, 33 and 35) was significantly different between IMU and KRT (FET, *p* = 0.03), with HPV16 in ~70% (23/33) of IMU and ~90% (46/51) of KRT, and all but one non-HPV16 type (HPV18) were in the IMU subtype, suggesting that HPV16 may be more successful at evading a host immune response. Age, stage and smoking status were not significantly different between IMU and KRT.

While KECK further divided the highly keratinized subgroup into basal (BA) and classical (CL) expression signatures, Locati, et al. divided this same group into high (Cl2) and low stromal (Cl3). These are associated with each other, as tumors having more basal features tend to have higher proportions of cancer-associated fibroblasts (CAFs), which constitute the majority of stromal cells [70]. This subset of KRT tumors may be more likely to exhibit a local invasion pattern, which enables the invading tumor cells to recruit CAFs, which evidence suggests can further promote invasion [71]. This may explain why high CAFs are associated with poor survival. With increasing data types and cohort sizes, more attributable factors will likely be identified to characterize the molecular subtypes in HPV(+) HNSCC, and finer-grained subtypes are expected to be defined.

## 8. Survival in Relationship to Subtypes and HPV Integration Status

There is increasing evidence that HPV subtypes and integration impact patient outcomes. For example, Locati et al., examined the relationship between three biological HPV(+) HNSCC subtypes and HPV(−)integration [23]. As described above, they identified three biologically distinct HPV(+) HNSCC subtypes, Cl1, Cl2 and Cl3. Specifically, Cl1 had enrichment of immune-related pathways, Cl2 overexpressed genes relating to EMT and high stromal content, and Cl3 was tied to proliferation and low stromal content. Notably, these subtype classifications are consistent with the HPV(+) HNSCC subtypes defined by microarray gene signatures described above. Furthermore, these subtypes also had different survival rates, which appear to be correlated with HPV integration. This may be attributed to distinct biological properties associated with HPV integration. For example, subgroup Cl1, which had the best prognosis (80.9% 5 year survival rate), had the least number of HPV(−)integrated cases whereas Cl2 had the highest percent of HPV integration and the worst survival outcome (19.7% 5 year survival rate) [23].

Many studies have examined how HPV integration impacts prognosis and survival of HPV positive head and neck cancer patients, focusing in particular on survival differences in patients with episomal versus integrated HPV. Early studies did not detect a significant difference in survival based on HPV integration status. In particular, a study of 179 patients with HPV(+) oropharyngeal cancer classified patients as integrated, episomal or mixed using the E2/E6 DNA copy number ratio, and found no significant differences in 3-year overall survival for HPV episomal versus integrated oropharyngeal squamous cell carcinoma patients, although patients with both integrated and episomal HPV genomes (i.e., mixed HPV), alluded to having better overall survival [72]. Limitations of this study, however, were having only 22 and 42 episomal and integrated patients, respectively, and misclassifying HPV integration-positive patients who retained high levels of E2. Likewise, another study evaluated 186 head and neck cancer patients and determined HPV integration status by mapping E2 at the mRNA level. They also did not detect a significant difference in disease-specific survival between patients with integrated, mixed or episomal HPV [73], although there was a trend for patients with the mixed and episomal form of HPV to have a better prognosis. Despite these early observations, later studies have reported significant findings. Nulton et al. [74] analyzed 56 HPV16(+) head and neck cancer patients and found that patients with integrated HPV head and neck cancers, in which HPV integration was defined by E2 and E7 gene expression, had a statistically significant reduction in survival compared to episomal HPV patients. They compared the 5-year survival rate and saw episomal HPV head and neck cancer patients had a survival rate of 72% whereas integrated HPV patients had a lower survival rate of 30% [74]. Notably, the survival rate of the integrated HPV head and neck cancer patients was also worse than the survival for patients with HPV(−) head and neck cancer, who had a survival rate of 40% [74]. Another RNAseq-based study that evaluated the impact of HPV integration on 84 HPV(+) head and neck cancers showed similar results with better survival in integration-negative patients than integration-positive [57]. In particular, both univariate and multivariate analyses showed HPV integration was associated with worse overall survival, and it was suggested that differences in survival between HPV integration positive and negative head and neck cancers may explain the survival variability in HPV(+) head and neck cancer patients.

The differences in survival in HPV(+) patients based on integration status may be attributed to the biological effects resulting from HPV integration. For example, RNAseq analysis of integration(−) samples had higher expression of immune related genes, including genes related to T cell and B cell activation. In contrast, integration(+) samples had higher expression of genes related to keratinization and RNA metabolism and translation that had also been previously noted in specific HPV(+) molecular subtypes [57]. These studies are promising for using HPV integration status as an additional prognostic marker that may dictate treatment approach. However, these results are still controversial. Recently published results saw improved survival in patients who had HPV integration, conflicting previously published studies [75]. One possible reason for the discrepancies is differences in how HPV is detected, as the relationship with survival may heavily depend on how integration is defined. Regardless, additional studies will need to be performed with larger sample sizes.

Survival and treatment response may also be affected by keratinization, which has been shown to cause cell death pathway alterations. High keratin levels in tumor cells have multiple potential mechanisms to resist apoptosis. Raj et al., reported that the HPV E1^E4 isoform protein typically binds to keratins, but in their absence or low expression, E1^E4 instead binds to mitochondria which causes mitochondrial detachment from microtubules, and a large decrease in the mitochondrial membrane potential [76]. This in turn induces apoptosis, suggesting that high levels of cytokeratin inhibit this chain of events leading to apoptosis. Keratinization may also have a more direct effect on cell survival by altering the shape and function of mitochondria [77]. In this case, these alterations may protect the cells from oxidative damage and other stressors, reducing apoptosis [78]. In terms of metastasis, high expression of one keratin, K17, resulted in an approximate 6-fold increase in lymph node metastasis. However, when accounting for treatment response, the relationship between keratins and survival may be more complex, as low levels of K17 were shown to correlate with worse prognosis for patients treated with surgery or radiotherapy. Treatment response may also be associated with keratinization as observed for multiple cancer types [79,80], depending on the complex interplay between treatment and cellular structural integrity, angiogenesis (which may affect the ability of oxygen to permeate the tumor and thus cellular oxidative damage) and the DNA damage response. Indeed, the Cl2 subtype (highly keratinized and high hypoxia) in Locati et al., had the highest radiosensitivity (RSI) score indicating high radioresistance [23].In Gleber-Netto et al., E1^E4 was found to positively correlate with radiation sensitivity, potentially explained by its negative correlation with mitochondrial genes suggesting reduced ability to respond to oxidative stress. In this study, E1^E4 had only a modest negative association with HPV integration status [81].

## 9. Future for De-Escalation Treatment in HPV-Positive Oropharynx Cancer Patients

Despite markedly better 5-year survival rates of HPV(+) oropharyngeal cancer patients compared to HPV(−) (~80% vs. 50%, respectively), treatment toxicity and quality of life (QoL) remain diminished, because standard HNSCC therapy is unnecessarily aggressive for many HPV(+) patients [82]. QoL deteriorations include dry mouth with difficulty chewing and/or swallowing (dysphagia), and speech difficulties [83]. However, two clinical trials assessing de-escalation of treatment for HPV(+) oropharyngeal cancer patients resulted in worse survival compared to standard treatment protocols [84], underscoring the importance of classifying HPV(+) patients into high- and low- risk cohorts, as well as predicting which subgroup of patients would most likely benefit from immune checkpoint inhibitor (e.g., anti-PD1/PD-L1) therapies. Thus, uniform, non-personalized de-escalated therapy involves unacceptable risk. Biomarkers identifying poor prognosis and/or predicting targeted treatment response in HPV(+) patients are lacking [85], with the exception of an immune score estimating infiltrating cytotoxic CD8+ T cells, B cells and regulatory T cells in the tumor [86,87,88]. E6 and E7 have been reported to be involved in the PI3K/AKT/mTOR regulatory network in both normoxic and hypoxic HPV(+) cancer cells [89], and thus these pathways are potential target pathways for alternative cancer therapies. E6 activates AKT, inducing a signaling cascade involving NFkB and cytokine IL-6, resulting in phosphorylation of Signal Transducer and Activator Of Transcription 3 (STAT3) which leads to cell survival and proliferation. E6 also activates mTORC1 which may serve to reduce autophagy, suggesting the potential use of mTORC1 inhibitors such as rapamycin in a subset of HPV(+) patients [90,91]. The most well-known effect of E6, activating p53 protein degradation, further transactivates Survivin, because p53 normally inhibits survivin expression. Survivin as a member of (Inhibitors of Apoptosis ) IAP gene family that is an essential regulator of cell division and protects cells from apoptosis [92,93], suggesting the possible use of IAP inhibitors (e.g., small molecular mimetics of SMAC) in a subset of HPV(+) patients with other indications of this pathway being overactivated [94].

While HPV(−)integration status has shown a tenuous relationship with survival, larger studies are needed, and biomarkers for the downstream effects will likely need to be taken into account in addition or in place of HPV integration status itself. This is due to the several carcinogenic pathways that are correlated, but not completely predictive, of HPV integration status. We conjecture that HPV(−)integration positive patients tend to have worse survival partly due to lower immunogenicity, and also potentially due to highly expressed keratins. In addition, the increased levels of the E6* spliced isoform observed in HPV(−)integration positive patients, which leads to higher E7 translation, may contribute to worse survival by altering the tumoral oxidative phosphorylation, oxidative stress and DNA damage. On the other hand, the higher EMT signatures in HPV(−)integration negative and IMU subgroup may lead to higher risk of distant metastasis, observed as the most common type of recurrence in HPV(+) oropharyngeal cancer [84]. Further investigations are necessary to disentangle the relative contributions of the various cancer pathway expression patterns to overall and disease-specific survival. Pathways likely to contribute and that differ by HPV(+) subtypes include tumor infiltrating lymphocyte levels, EMT and mesenchymal differentiation signatures, keratinization, cell cycle regulation and DNA damage response. This suggests the potential for using HPV(+) subtypes to identify patients at high risk and who are good candidates for alternative therapies including anti-PD-1 [95,96,97] or PI3K inhibitors [98]. Ultimately, classification of HPV(+) patients into two or three subgroups will remain an oversimplification of the complex, multi-dimensional signatures that together determine the prognosis and treatment response of each patient. Until the field reaches that advanced level of knowledge, however, understanding the characteristics of the major subtypes and their relationship with recurrence and survival will hopefully lead us to patient subgroups most likely to benefit from de-escalated therapy, ICI therapy or another targeted treatment.

## Figures and Tables

**Table 1:** Gene Expression Studies that Defined HPV(+) HNSCC Subtypes.

Citation	HPV(+) Subtype Names	Figure Abbr	Data Used to Define Subtypes	Sample Size	Sites
Pyeon et al., 2007	α (B cell strong), β (highly keratinized)	-	Expression microarrays	16 HPV(+); 26 HPV(−)	Oropharynx; oral cavity
Keck et al., 2015	IMS (immune strong), BA (basal-like), CL (classical)	KECK	Expression microarrays	371 total; 55 HPV(+); 75 HPV(−) *	Oropharynx; oral cavity; larynx
TCGA 2015	Atypical, Basal, Classical, Mesenchymal	NAT	RNAseq	36 HPV(+); 243 HPV(−)	All HNSCC
Zhang et al., 2016	IMU (immune strong); KRT (highly keratinized)	CCR	RNAseq	84 HPV(+); 18 HPV(−)	Oropharynx; oral cavity
Lee et al., 2018	1 (cervical-like); 2 (HNSCC classical); 3 (lung-like)	ORA	RNAseq	1346 total; 514 HNSCC; 65 HPV(+)	HNSCC, esophageal, lung, cervical
Locati et al., 2019	Cl1 (immune strong, high stromal); Cl2 (highly keratinized; high stromal); Cl3 (highly keratinized; low stromal)	CAN	RNAseq and microarrays meta-analysis	346 HPV(+)	Oropharynx, oral cavity, larynx, hypopharynx

* For Keck et al., only 130 have known HPV status.

## Data Availability

The data presented in this study is openly available in reference numbers [21,23,24]. Publicly available datasets were analyzed in this study. This data can be found here: [https://portal.gdc.cancer.gov/, accessed on 30 April 2021, and Gene Expression Omnibus #GSE74956].

## Supplementary Materials

The following are available online at https://www.mdpi.com/article/10.3390/cancers13112721/s1, Figure S1: Comparisons of pathway scores among the 84 HPV(+) HNSCC samples, which were stratified by IMU/KRT subtypes and HPV integration status. Figure S2: Comparisons of average Fanconi Anemia (FA) genes among the 84 HPV(+) HNSCC samples.
