# Supplementary material for: Molecular Tumor Subtypes of HPV-Positive Head and Neck Cancers: Biological Characteristics and Implications for Clinical Outcomes
Source: Cancers (Basel). 2021 May 31;13(11):2721. doi: 10.3390/cancers13112721 (PMC8198180; doi:10.3390/cancers13112721)
Supplement: Supplementary file 1 [file cancers-13-02721-s001.zip › cancers-1225293-supplementary.pdf]

cancers

MDPI

Review

Molecular Tumor Subtypes of HPV-Positive Head and Neck Cancers: Biological Characteristics and Implications for Clinical Outcomes

Tingting Qin [1,2,†], Shiting Li [1,†], Leanne E. Henry [1,3], Siyu Liu [1] and Maureen A. Sartor [1,2,*]

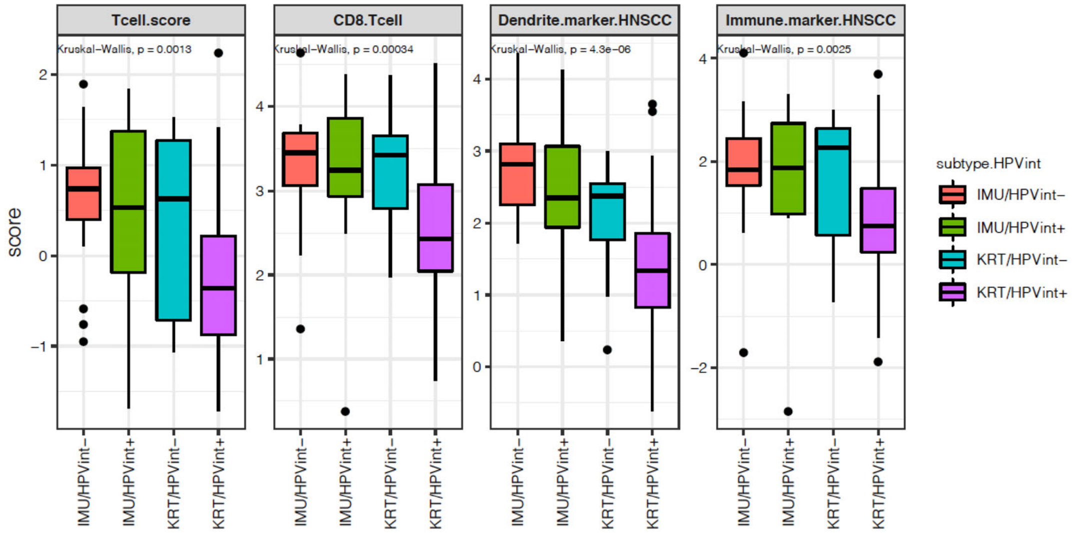

Supplementary Figure S1. Comparisons of pathway scores of T cell differentiation (Tcell.score), CD8 T cell (CD8.Tcell), Dendrite cell marker for head neck cancer (Dentrite.marker.HNSCC) and Immune Marker for Head Neck Cancer (Immune.marker.HNSCC) among the 84 HPV(+) HNSCC samples from combined TCGA and UM cohorts, which were stratified by IMU/KRT subtypes and HPV integration status.

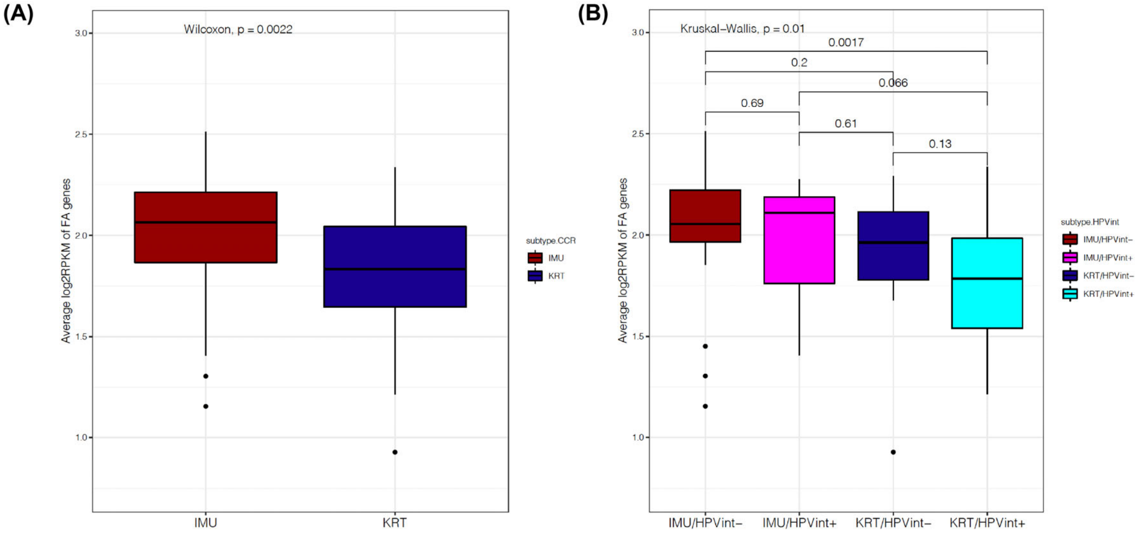

Supplementary Figure S2. Comparisons of average fanconi anemia (FA) genes among the 84 HPV(+) HNSCC samples from combined TCGA and UM cohorts between (A) IMU/KRT subtypes and (B) IMU/KRT subtypes and HPV integration status.
